# Numerical Study of Flow and Heat Transfer Characteristics for Al_2_O_3_ Nanofluid in a Double-Pipe Helical Coil Heat Exchanger

**DOI:** 10.3390/mi14122219

**Published:** 2023-12-09

**Authors:** Hyeon Taek Nam, Sumin Lee, Minsuk Kong, Seungro Lee

**Affiliations:** 1 Department of Mechanical Engineering, Jeonbuk National University, 567 Baekje-daero, Deokjingu, Jeonju-si 54896, Jeollabuk-do, Republic of Korea; skagusxor@jbnu.ac.kr (H.T.N.); sumin2918@hanyang.ac.kr (S.L.); 2 Department of Equipment and Fire Protection Engineering, Gachon University, 1342 Seongnam-daero, Sujeong-gu, Seongnam-si 13120, Gyeonggi-do, Republic of Korea; 3 Laboratory for Renewable Energy and Sector Coupling, Jeonbuk National University, 567 Baekje-daero, Deokjin-gu, Jeonju-si 54896, Jeollabuk-do, Republic of Korea

**Keywords:** Al_2_O_3_ nanofluid, double-pipe helical coil heat exchanger, two-phase Eulerian model, Nusselt number, pressure drop, performance factor

## Abstract

To numerically investigate the flow and heat transfer characteristics of a water/Al_2_O_3_ nanofluid in a double-pipe helical coil heat exchanger, we simulated a two-phase Eulerian model to predict the heat transfer coefficient, Nusselt number, and pressure drop at various concentrations (i.e., volume fraction) and under diverse flow rates at the steady state. In this simulation, we used the k-epsilon turbulence model with an enhanced wall treatment method. The performance factor of the nanofluid was evaluated by accounting for the heat transfer and pressure drop characteristics. As a result, the heat transfer was enhanced by increasing the nanofluid concentration. The 1.0 vol.% nanofluid (i.e., the highest concentration) showed a heat transfer coefficient 1.43 times greater than water and a Nusselt number of 1.38 times greater than water. The pressure drop of nanofluids was greater than that of water due to the increased density and viscosity induced using nanoparticles. Based on the relationship between the Nusselt number and pressure drop, the 1.0 vol.% nanofluid was calculated to have a performance factor of 1.4 relative to water, indicating that the enhancement rate in heat transfer performance was greater than that in the pressure drop. In conclusion, the Al_2_O_3_ nanofluid shows potential as an enhanced working fluid in diverse heat transfer applications.

## 1. Introduction

An enhanced heat transfer performance is required to develop new thermal management systems. Diverse means of achieving high heat transfer performance have been suggested to employ various enhancing techniques, including passive, active, and compound techniques. The passive technique enhances the heat transfer performance without extra power applications. For example, it has been suggested that the surface area can be increased by making it more rough (e.g., through fabrication of micro/nano-structures [1,2,3,4,5]) and using new working fluids that mix additives or nano-sized particles in base fluids [6,7,8,9,10,11,12]. Wen et al. [1] investigated the boiling heat transfer using nanowire structures. It was found that the nanowire structures had a capillary-induced re-wetting property which enhanced the heat transfer performance. The capillary wicking was induced through a surface modification. This capillary wicking is well known as a crucial factor in determining the boiling heat transfer performance due to the critical heat flux owing to the enhanced fluid supply into the heating surface [2]. Li et al. [3] examined the flow boiling heat transfer by using wicked-microchannel structures. They found that the wicked-microchannel had an enhanced critical heat flux by up to 1.8 times, and a heat transfer coefficient of up to 75% due to the high-wetting performance of wicking. Schell et al. [4] fabricated laser-textured microstructures using the Direct Laser Interference Patterning (DLIP) method to enhance the heat dissipation performance of a heat sink. They found that the laser-textured microstructures improved heat dissipation by approximately 51.4% compared to the non-textured sample due to its increased surface area. Nam et al. [5] used the Micro Electro Mechanical Systems (MEMSs) process to fabricate micro-pillar structures. The narrow-gap micro-pillar structures had a roughened surface, enhancing the heat transfer performance. In particular, the micro-pillar structures enhanced the wicking performance induced through capillary force between the structures, resulting in an enhanced boiling heat transfer by promoting the fluid supply capacity. For the additive method, Mahmoudi et al. [6] investigated convective heat transfer using a TiO_2_/water nanofluid and found that the nanofluid led to an enhancement in the Nusselt number by up to 30% compared to pure water. Sahin et al. [7] experimentally examined the heat transfer characteristics of Al_2_O_3_/water nanofluid with various volume fractions (i.e., *φ* = 0~4 vol.%). Based on their results, the concentrations of the nanofluid that were higher than 1 vol.% were not suitable for heat transfer enhancement due to their increased viscosity and friction factor. Heyhat et al. [8] investigated the turbulent flow and convective heat transfer performance of Al_2_O_3_/water nanofluid in a circular tube heat exchanger. The heat transfer coefficient of nanofluid was higher than that of the base fluid (i.e., water), with a volume fraction of 0.1–2 vol.%. Nasiri et al. [9] experimentally investigated the heat transfer of Al_2_O_3_/water and TiO_2_/water nanofluids through an annular duct heat exchanger. Both the Al_2_O_3_/water and TiO_2_/water nanofluids showed an enhanced and similar heat transfer coefficient and Nusselt number compared to the base fluid due to their high thermal conductivity. Vajjha et al. [10] developed a new correlation for the convective heat transfer and friction factor for various nanofluids from experiments. As the concentration increased, the heat transfer coefficient increased. However, the pressure drop increased with the nanofluid concentrations due to the increased viscosity. Wai et al. [11] reviewed the many investigations of jet impingement cooling performance with nanofluids. They reported that Al_2_O_3_/water nanofluids are the most used working fluids in the experimental approaches due to their widespread industrial applicability. Bouselsal et al. [12] numerically investigated the heat transfer enhancement using an Al_2_O_3_-MWCNT/water hybrid nanofluid in a tube/shell heat exchanger. They found that the nanofluids had an enhanced heat transfer with a growing nanoparticle concentration. The 2% nanofluid showed a higher heat transfer performance of up to 103% than pure water. The active technique or compound technique (combination of passive and active techniques) applies an extra power device (e.g., using surface vibration, jet impingement, electric/magnetic fields, etc.) [13]. These techniques enhance the heat transfer performance more than the passive approach, but they are more costly. For this reason, the passive approach is more widely used.

Heat exchangers with a large surface area accelerate the heat transfer performance. A coil-tube heat exchanger improves the heat transfer due to its large surface area per occupied volume. Accordingly, this can be considered the most suitable heat exchanger for developing future thermal systems that improve heat dissipation. Notably, the centrifugal force induced through the coil curvature generates a secondary flow (i.e., flow disturbance) inside the pipe, resulting in a higher heat transfer rate than a straight pipe. For this reason, research on heat transfer with a coil tube is ongoing. Many researchers have investigated the heat transfer characteristics and pressure drops using various design parameters (e.g., tube types, coil diameters, pitches, and turns) [14,15,16]. A coiled-tube heat exchanger consists of shell-and-coil and double-pipe helical types. The shell-and-coil heat exchanger carries with it the high possibility of a dead zone—a region where heat transfer does not occur owing to the non-flow of fluid—in the flow field due to its complex structure. This dead zone causes an unstable heat transfer, reducing the thermal efficiency. In contrast, a double-pipe helical coil heat exchanger prevents such zones because its surface area is in perfect contact with the working fluid. Moreover, an additional secondary flow driven by the curved annular tube can be generated, which further enhances the heat transfer performance.

Heat transfer can also be enhanced by improving the thermal conductivity of the working fluids. For example, new working fluids (i.e., nanofluids), made by mixing nano-sized metallic particles (i.e., nanoparticles) with a high thermal conductivity with traditional base fluids (e.g., water, ethylene glycol, oil, etc.), have the potential to enhance the heat transfer. The nanofluid suggested by Choi and Eastman [17] has good thermal properties (i.e., thermal conductivity) and dissipation stability. Many researchers have evaluated the thermal properties using various nanofluids [6,7,8,9,10,18], and have investigated relevant heat transfer and flow characteristics using the nanofluid properties with various heat exchanger shapes and operating conditions. However, an accurate evaluation of enhanced heat transfer using nanofluids can be conducted only through proper experiments (e.g., nanofluid fabrication with a uniform distribution and visualization setup for the flow phenomenon). Due to the difficulty of the experimental approach, it is necessary to perform a numerical study on the heat transfer of nanofluids.

Numerical research about nanofluids must first consider the nanofluid as either a single-phase or multi-phase (i.e., solid–liquid). The single-phase approach treats the nanofluid as a single fluid, generating results that can be more easily predicted than the multi-phase approach. In a previous study [19,20], it was found that a numerical approach assuming the single-phase state accurately predicted heat transfer characteristics. However, the actual application of nanofluids may require a two-phase analysis because it is vital to understand particle behavior (e.g., particle distribution) within the flow field. To date, two-phase numerical studies on the heat transfer characteristics of nanofluids have been mainly conducted using a simple configuration (e.g., microchannel [21], singe helical coil [22,23], annulus [24], standard tube [25,26], and its simplified domain [27,28,29]). For complex types, such as double-pipe helical coil heat exchangers, numerical studies have been conducted by assuming the single-phase [30] or applying laminar flow ranges [31]. To overcome these limitations, this study numerically investigated the heat transfer and hydraulic characteristics of a double-pipe helical coil heat exchanger for a water/Al_2_O_3_ nanofluid, which has been well known to have a high thermal conductivity and stability [32], at various concentrations (i.e., volume fraction, φ~1.0 vol. %) using a two-phase Eulerian model. In addition, the energy efficiency was evaluated using a performance factor determined by reference to both heat transfer and pressure drop characteristics.

## 2. Numerical Methods

### 2.1. Two-Phase Model

The two-phase numerical model applied either a Euler–Lagrangian or Euler–Euler approach. In the Euler–Lagrangian approach, the fluid was considered as the continuum. The particles in the fluid were considered a separate phase and were tracked during simulation. Mass, momentum, and energy are transferred to each other in the continuum and between particles. In the Euler–Lagrangian approach, a high-performance workstation is required to simulate the many nanoparticles in the calculation domain [19]. Meanwhile, the Euler–Euler approach considers each phase (i.e., particle or fluid) as an interpenetrating continuum passing the calculation domain. The volume fraction of one phase is not occupied in that of other phases. In this study, we employed the Euler–Euler approach to predict the heat transfer of the nanofluids using commercial software (ANSYS Fluent 18.1 [33]). ANSYS Fluent software provides three Euler–Euler multi-phase models: Volume of fluid (VOF), Mixture, and Eulerian. The VOF model uses the surface tracking technique in the fixed grids. This model is mainly used for stratified flow and free-surface motion. The mixture model calculates the mixture momentum equation using the relative velocities for each phase. The particle-laden flows and bubbly flows are calculated with this mixture model. In the Eulerian model, each phase is calculated separately using the governing equations (i.e., continuity, momentum, energy). The linkage between phases is achieved by pressure and exchange coefficient depending on the kinds of phase (i.e., fluid–solid and solid–solid) [34]. In light of the features of the Eulerian model, we employed the Eulerian model to simulate the heat transfer characteristics of nanofluids in a double-pipe helical coil heat exchanger.

The governing equations were solved for each phase (i.e., base fluid and particle) [33]. The continuity equation (i.e., mass conversion) is shown as Equations (1)–(3):

(1) $$\nabla \cdot \left(\varphi_{l}\rho_{l}{\vec{v}}_{l}\right)=0$$

(2) $$\nabla \cdot \left(\varphi_{s}\rho_{s}{\vec{v}}_{s}\right)=0$$

(3) $$\varphi_{l}+\varphi_{s}=1$$

in which the l and s subscripts represent the liquid and solid phases, respectively.

The momentum equation for liquid phase (l) was

(4) $$\nabla \cdot \left(\varphi_{l}\rho_{l}{\vec{v}}_{l}{\vec{v}}_{l}\right)=-\varphi_{l}\nabla P+\nabla \cdot \overset{=}{\tau_{l}}+\varphi_{l}\rho_{l}\vec{g}+\sum_{s=1}^{n\;}{\vec{R}}_{sl}+\left({\vec{F}}_{l}+{\vec{F}}_{lift,l}+{\vec{F}}_{vm,l}\right)$$

where P means the shared pressure on all phases. ${\vec{F}}_{l}$, ${\vec{F}}_{lift,l}$, and ${\vec{F}}_{vm,l}$ represent the external body force, lift force, and virtual mass force, respectively. The viscous stress tensor, $\overset{=}{\tau_{l}}$, can be expressed using Equation (5).

(5) $$\overset{=}{\tau_{l}}=\varphi_{l}\tau_{l}\left(\nabla {\vec{v}}_{l}+\nabla {\vec{v^{T}}}_{l}\right)+\varphi_{l}\left(\lambda_{l}-\frac{2}{3}\mu_{l}\right)\nabla \cdot {\vec{v}}_{l}\overset{=}{I}$$

The interphase momentum force (i.e., the volume force induced on each phase by the other),$\;{\vec{R}}_{sl}$, was determined using Equation (6).

(6) $$\sum_{s=1}^{n\;}{\vec{R}}_{sl}=\sum_{s=1}^{n\;}K_{sl}\left({\vec{v}}_{s}-{\vec{v}}_{l}\right)$$

in which $K_{sl}$ represents the interface momentum transfer coefficient. The momentum equation for the solid phase (s) was

(7) $$\begin{array}{ll} \nabla \cdot \left(\varphi_{s}\rho_{s}{\vec{v}}_{s}{\vec{v}}_{s}\right) & =-\varphi_{s}\nabla P+\nabla P_{s}+\nabla \cdot \overset{=}{\tau_{s}}+\varphi_{s}\rho_{s}\vec{g}\;\\ & +\sum_{l=1}^{N\;}K_{ls}\left({\vec{v}}_{l}-{\vec{v}}_{s}\right)+\left({\vec{F}}_{s}+{\vec{F}}_{lift,s}+{\vec{F}}_{vm,s}\right) \end{array}$$

where $P_{s}$ represents the solid pressure induced by an impingement between the particles. The interface momentum transfer term in the momentum equation refers to the drag force. Interface momentum transfer coefficients ($K_{sl}$ and $K_{ls})$ were determined using the Wen and Yu model (see Equations (8)–(10) [35]):

(8) $$K_{sl}=K_{ls}=\frac{3}{4}C_{D}\frac{\varphi_{s}\varphi_{l}\rho_{l}\left|{\vec{v}}_{s}-{\vec{v}}_{l}\right|}{d_{s}}\varphi_{l}{}^{-2.65}$$

(9) $$C_{D}=\frac{24}{\varphi_{l}Re_{s}}\left[1+0.15{\left(\varphi_{l}Re_{s}\right)}^{0.687}\right]$$

(10) $$Re_{s}=\frac{\rho_{l}d_{s}\left|{\vec{v}}_{s}-{\vec{v}}_{l}\right|}{\mu_{l}}$$

The lift force (${\vec{F}}_{lift}$) was neglected due to the significant small (nano-) size of particles [19,21]. The virtual mass effect occurs when the solid phase accelerates faster than the liquid phase (see Equation (11)).

(11) $${\vec{F}}_{vm}=C_{vm}\varphi_{s}\rho_{l}\left(\frac{d{\vec{v}}_{l}}{dt}-\frac{d{\vec{v}}_{s}}{dt}\right)$$

The virtual mass coefficient ($C_{vm}$) was 0.5, treating the particle shape as a sphere.

The energy equation was as follows.

(12) $$\nabla \cdot \left(\varphi_{q}\rho_{q}{\vec{v}}_{q}h_{q}\right)=\overset{=}{\tau_{q}}∶\nabla {\vec{u}}_{q}-\nabla \cdot {\vec{q}}_{q}+\sum_{p=1}^{n}Q_{pq}$$

where $h_{q}$ and ${\vec{q}}_{q}$ represent the specific enthalpy and heat flux on the q-phase, respectively. $Q_{pq}$ means the heat transfer rate between the *p*–*q* phases, as calculated using Equations (13) and (14).

(13) $$Q_{pq}=h_{pq}A_{i}\left(T_{p}-T_{q}\right)$$

(14) $$h_{pq}=\frac{k_{q}Nu_{p}}{d_{p}}$$

where $h_{pq}$ and $k_{q}$ represent the heat transfer coefficient between the *p*–*q* phases and the thermal conductivity of the *q*-phase, respectively. The Nusselt number of the p-phase ($Nu_{p}$) was calculated using the Ranz and Marshall model (see Equation (15) [36]).

(15) $$Nu_{p}=2+0.6Re_{p}^{1/2}Pr^{1/3}$$

### 2.2. Simulation Geometry and Boundary Conditions

**Figure 1:**
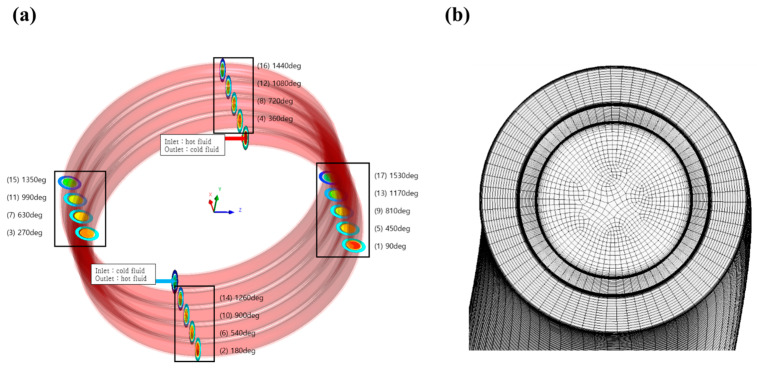
(**a**) Geometry and (**b**) mesh of double-pipe helical coil heat exchanger.

To investigate the heat transfer characteristics of the Al_2_O_3_ nanofluid in a double-pipe helical coil heat exchanger, we simulated the two-phase Eulerian model to predict the heat transfer coefficient, Nusselt number, and pressure drops using the three-dimensional flow domain. For the simulation, we calculated the heat transfer and hydraulic characteristics of the Al_2_O_3_ nanofluid at a steady state. Figure 1a depicts the three-dimensional geometry of a double-pipe helical coil heat exchanger. The hot fluid (i.e., 30 °C) and the cold fluid (i.e., 20 °C) were introduced to the center and annulus tubes, respectively. The nanofluids flowed through the hot side and water flowed through the cold side. The two fluids flowed as the counter flow. The detailed size of the heat exchanger is described in Table 1. The outer wall of the annulus tube (i.e., cold side) was assumed to be adiabatic. Table 2 shows the properties of Al_2_O_3_ nanofluid components (i.e., particle and water) used in this study. Since the water properties change depending on temperatures, a third-order polynomial equation was used to reflect the accurate thermal properties during the heat transfer [37]. Table 3 shows the coefficients of polynomial equations for thermal properties depending on water temperatures. Table 4 and Table 5 show the numerical methods and conditions for the present study. The walls of the center and annulus tubes were applied as no-slip boundary conditions. At the inlet, uniform flow velocity and temperature were employed ($u_{inlet}=u_{0},\;T_{inlet}=T_{0}).$ The flow rate on the hot side was set to 3.2~4.8 LPM while the flow rate on the cold side was fixed to 7.2 LPM. The outlets of the hot and cold sides were applied to the zero-pressure condition ($P_{outlet}=0$).

### 2.3. Validation of Simulation 

#### 2.3.1. Mesh Independence Test

**Figure 2:**
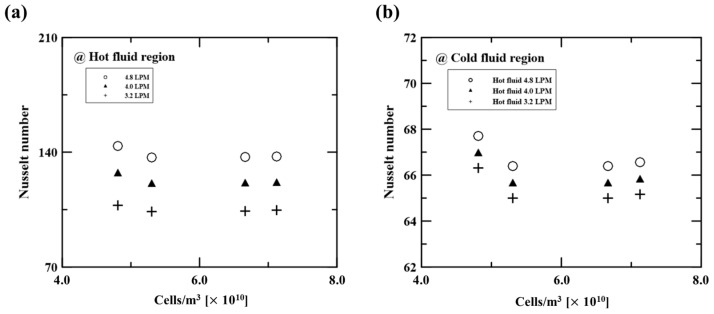
Nusselt number depending on the various cell number cases. (**a**) Nusselt number in the hot side depending on the mesh cases. (**b**) Nusselt number in the cold side depending on the mesh cases.

Figure 1b depicts the mesh in the calculation domain of the heat exchanger. This mesh case was selected by conducting mesh independence tests. We compared the calculated Nusselt number by changing the Y^+^ values on the hot side. The dense grid near the wall (i.e., Y^+^~1.0) was needed to reflect the rapid gradient of velocities and temperatures. The four mesh cases used in the mesh independence tests are described in Table 6. The working fluid in this test was water. Figure 2 shows the predicted Nusselt number with each mesh case. Mesh case 1, which had the highest Y^+^ value (6.50), overestimated the Nusselt number relative to other cases. As the Y^+^ value approached 1.0, the Nusselt number converged to a constant value. Mesh cases 3 and 4 showed nearly identical results, with a difference of 0.5%. Based on this finding, we selected mesh case 3 (Y^+^ = 1.35).

#### 2.3.2. Model Validation

In this section, we validated the two-phase Eulerian model by comparing the predicted results with previously suggested correlations [38,39]. The simulations were conducted using a single tube with a diameter of 12.573 mm at a Reynolds number of 11,000~16,000. The working fluids were water and 0.81 vol.% Al_2_O_3_ nanofluid. The Nusselt number correlation of the water was suggested by Petukhov [38], as shown in Equations (16) and (17):

(16) $$\mathrm{Nu}=\frac{(f/8)RePr}{1.07+12.7{\left(f/8\right)}^{0.5}\left(Pr^{2/3}-1\right)}$$

(17) $$f={\left(1.82\log Re-1.64\right)}^{-2}\;$$

The correlation of 0.81 vol.% Al_2_O_3_ nanofluid was suggested by Maiga [39] as

(18) $$\mathrm{Nu}=0.085Re^{0.71}Pr^{0.35}\;$$

**Figure 3:**
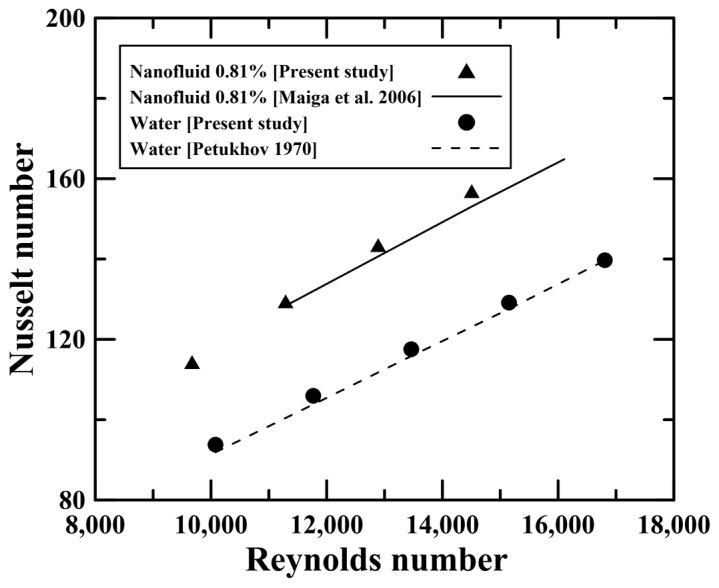
Nusselt number in previous correlations [38,39] and the present study.

Figure 3 compares previously suggested correlations with the predicted Nusselt numbers from the present simulation. In the case of water, it was found that the two-phase Eulerian model had a small difference (i.e., 2.12%) from the Petukhov correlation. The Nusselt number of the 0.81 vol.% Al_2_O_3_ nanofluid also showed a small difference (i.e., 2.51%) from the Maiga correlation. Based on these findings, the two-phase Eulerian model could be expected to accurately predict the heat transfer characteristics of nanofluids.

## 3. Results and Discussion

### 3.1. Flow and Temperature Distributions in Double-Pipe Helical Coil Heat Exchanger

**Figure 4:**
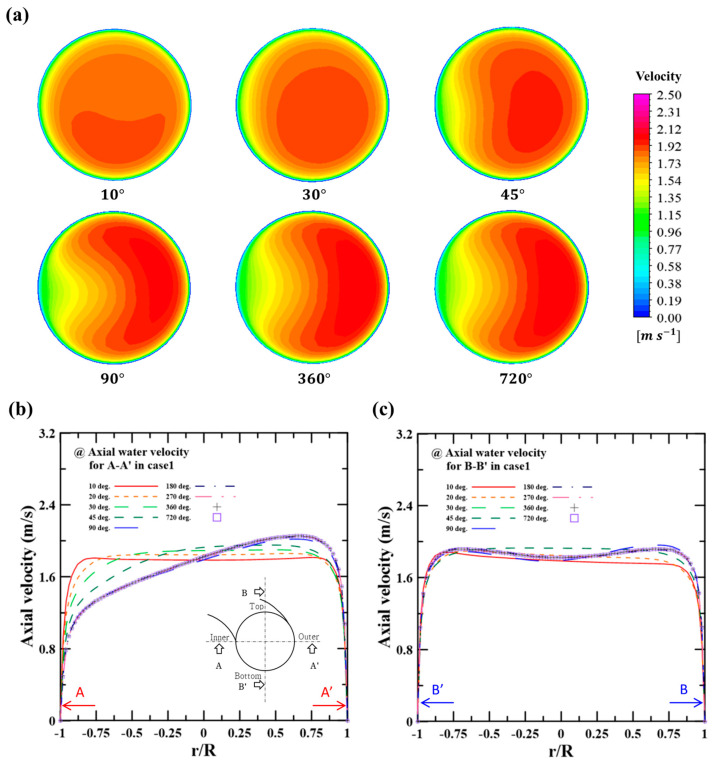
(**a**) Velocity contours along the coil length, (**b**) axial velocity profiles on the A-A’ crossline, and (**c**) axial velocity profiles on the B-B’ crossline on the hot side.

The flow in the double-pipe helical coil heat exchanger experienced significant disturbance along the coil length due to the centrifugal force induced by the coil curvature. This flow disturbance became dominant before a fully developed region, causing irregular flow and complicating the prediction. Therefore, it was necessary to find a fully developed region in which the thermal characteristics of the double-pipe helical coil heat exchanger could be examined. Figure 4 shows the flow velocity contours and profiles along the coil lengths (i.e., degrees) of the heat exchanger in case 1 (cold side: 7.2 LPM, hot side: 4.8 LPM). Figure 4a shows the velocity contours along the various coil lengths (from 10 to 720 degrees). As shown in Figure 4a, it was found that the velocity distribution became similar after 360 degrees. Figure 4b,c depict the velocity profiles on the A-A’ crossline (inner and outer) and on the B-B’ crossline (top and bottom) of the hot side. The inset of Figure 4b shows the position of the A-A’ and B-B’ crosslines. The flow velocity was biased toward the point where r/R is 1 (i.e., outer region) at the A-A’ crossline, as the flow momentum inertia was significant in the outer region due to the rotational flow in the coiled tube. These non-parabolic velocity profiles were uniform from one rotation (360 degrees). The velocities after one rotation were similar to those after two rotations (720 degrees). The velocity profiles on the B-B’ crossline were also constant after two rotations (720 degrees). Accordingly, the analysis of the heat transfer and hydraulic characteristics was performed after the 720-degree position to consider the fully developed region.

**Figure 5:**
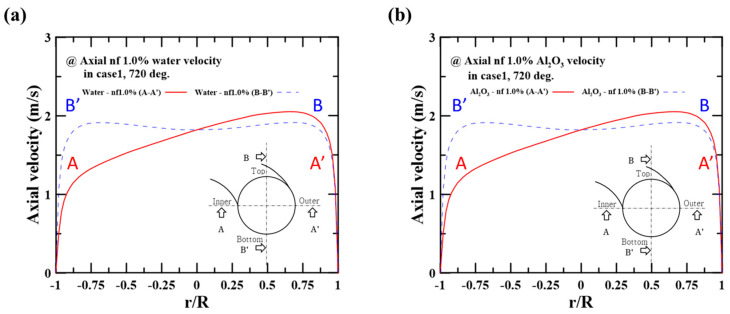
Axial velocity profiles on the A-A’ crossline and B-B’ crossline using 1.0 vol.% nanofluid: (**a**) base fluid (water) velocity and (**b**) particle velocity.

The two-phase Eulerian model calculates the governing equations for each phase (i.e., liquid and solid phases) to predict the velocities and temperatures of the particle or the base fluid. To analyze the two-phase heat transfer in the nanofluids, the interaction between the particle and the base fluid must be examined. For example, if the velocities of particles and water differ, a shear force results from the velocity gradient at the particle interface. We therefore compared the velocities of the solid phase (i.e., nanoparticle) and liquid phase (i.e., water) to predict the velocity-driven shear force at the particle interface. Figure 5a,b depict the velocity profiles of the base fluid (i.e., water) and the particle (i.e., Al_2_O_3_) on the hot side. The 1.0 vol.% nanofluid was used as the working fluid. In Figure 5, the velocities were obtained after two rotations (i.e., after a fully developed region). The velocities of water and the Al_2_O_3_ particle were observed to be biased toward the outer region (i.e., r/R~1 at A-A’ crossline). Simultaneously, the velocities of water and the Al_2_O_3_ particle were nearly the same, with a difference of less than 10^−6^ m/s. This result means that the velocity gradient did not occur between the base fluid and particles, i.e., there was only a small amount of shear force. 

**Figure 6:**
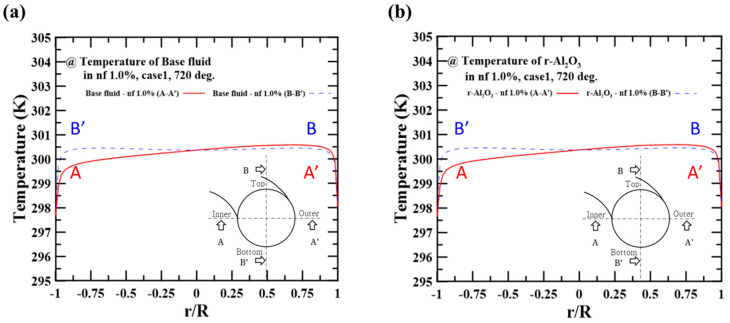
Temperature distribution on the A-A’ crossline and B-B’ crossline using 1.0 vol.% nanofluid: (**a**) base fluid (water) temperature and (**b**) particle temperature.

Figure 6a,b show the temperature profiles of the base fluid and the particle at the hot side. As shown in Figure 6a, the temperature distribution was also biased toward the outer region (i.e., r/R~1 at A-A’ crossline). This finding can be explained through reference to the velocity distribution. At the fixed flow rate condition on the cold side, the low velocity near the hot side’s inner region (i.e., r/R~−1 at A-A’ crossline) caused significant heat dissipation to the cold side, lowering the temperature. Figure 6b also shows that the particle temperatures had similar values to the fluid temperatures; the base fluid showed an average temperature of 300.8 K while the Al_2_O_3_ particle showed a temperature of 300.5 K. This result means that the particle interface in the nanofluid was unaffected by the heat transfer. Accordingly, the results predicted at the present numerical condition (i.e., flow rate and nanofluid concentration) did not have to account for the interface’s shear force and heat transfer.

### 3.2. Heat Transfer Characteristics of Nanofluids

**Figure 7:**
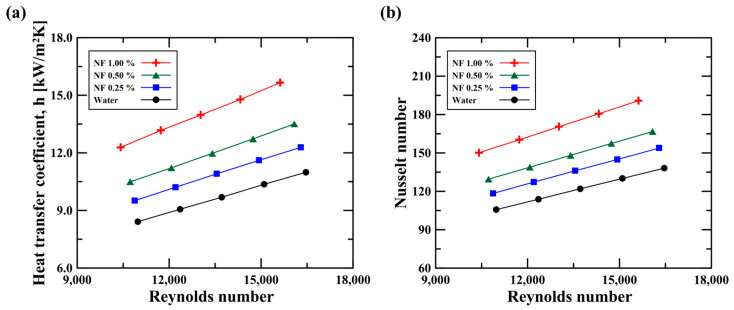
(**a**) Heat transfer coefficient and (**b**) Nusselt number on the hot side depending on nanoparticle concentration.

Figure 7a,b show the heat transfer coefficient and the Nusselt number at the hot side (i.e., center tube), where the nanofluid passes. The x-axis represents the Reynolds number. The Reynolds number (Re) was calculated as follows:

(19) Re=ρuDhμ

where $D_{h}$ refers to the hydraulic diameter and has a value of 0.00774 m. The heat transfer coefficient was determined by calculating the surface-averaged heat transfer coefficient after the fully developed region. The Nusselt number (Nu) was calculated as follows:

(20) $$Nu=\frac{hD_{h}}{k}$$

Figure 7a shows that the heat transfer coefficient increased with the Reynolds number. The high flow rate (i.e., high Reynolds number) promoted convective heat transfer, resulting in a raised heat transfer coefficient due to the increased momentum disturbance and enhanced turbulence effect in the curved pipe. For water during the 4.8 LPM (Re 16,400) condition, the heat transfer coefficient increased by 1.3 times relative to the 3.2 LPM (Re 11,000). Likewise, the heat transfer coefficient rose as the concentration of the nanofluid increased. At the 1.0 vol.% nanofluid (i.e., the highest concentration), the heat transfer coefficient was 15.7 kW/m^2^∙K, which was 1.43 times greater than that of water. This enhanced heat transfer can be explained by the significant cooling capacity with high thermal conductivity, and implies that nanofluids could improve thermal performance even under low flow rate conditions. As shown in Figure 7b, the enhanced heat transfer coefficient of the nanofluid increased the Nusselt number, which determines the convective heat transfer performance. The 1.0 vol.% nanofluid showed a Nusselt number of 191, which was 1.38 times higher than that of water. An enhanced Nusselt number means that the convective heat transfer coefficient increased more compared to the thermal conductivity, as shown in Equation (20). Based on these findings, it was found that the Al_2_O_3_ nanofluid had an enhanced convective heat transfer performance.

### 3.3. Pressure Drop

**Figure 8:**
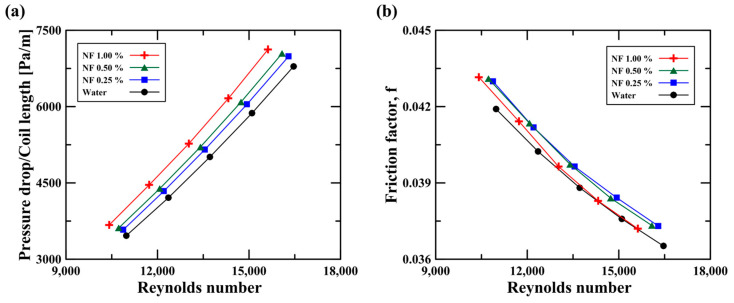
(**a**) Pressure drop per coil length and (**b**) friction factors on the hot side for the Reynolds number.

The application of nanofluids to heat exchangers must also account for heat transfer as well as the hydraulic characteristics (i.e., pressure drop), which defines pumping power. A pump system would have to be newly designed to reflect the change in working fluids for normal operations. We therefore analyzed pressure drops under various conditions (i.e., flow rates and concentrations). Figure 8a,b depict the pressure drops and friction factors at various flow rates and concentrations. The pressure drop rapidly increased with the Reynolds number (i.e., flow rate). The large flow rate (i.e., high velocity) proportionally affected the increasing pressure drop (i.e., $\Delta P\propto u^{2})$. When the water flow rate increased from 3.2 LPM to 4.8 LPM, the pressure drop rose from 3460 Pa/m to 6790 Pa/m. This is because of the large dynamic pressure loss and enhanced turbulence driven by the high fluid velocity. The pressure drop also increased as the concentration rose, since the fluid density and viscosity increased with the nanofluid concentration. As shown in Table 2, the Al_2_O_3_ nanoparticle had a high density of 3970 kg/m^3^. Previous studies have evaluated the viscosity of nanofluids and confirmed that their viscosity increases with their concentration [39,40]. Based on these results, we inferred that the Al_2_O_3_ nanofluid had a high pressure drop due to the increased flow resistance driven by the high fluid density and viscosity. In particular, the enhanced viscosity of the nanofluid promoted the viscous shear stress near the wall, resulting in the increased flow resistance and large pressure drop. Figure 8b shows the friction factors with various flow rates and concentrations. The friction factor was calculated as follows:

(21) $$f=\frac{D_{h}\Delta P}{2L\rho u^{2}}$$

As shown in Figure 8b, it was found that the friction factor at the 1.0 vol.% nanofluid was lower than that for the 0.25 vol.% nanofluid. Although there was a low friction factor at the high concentration, the pressure drop increased due to the high fluid density and viscosity. Therefore, an increased concentration of nanofluids would contribute to the rise in pressure drop due to the enhanced flow resistance owing to the increased fluid density and viscosity (i.e., ΔP∝ρ,μ).

### 3.4. Performance Evaluation of Nanofluids

**Figure 9:**
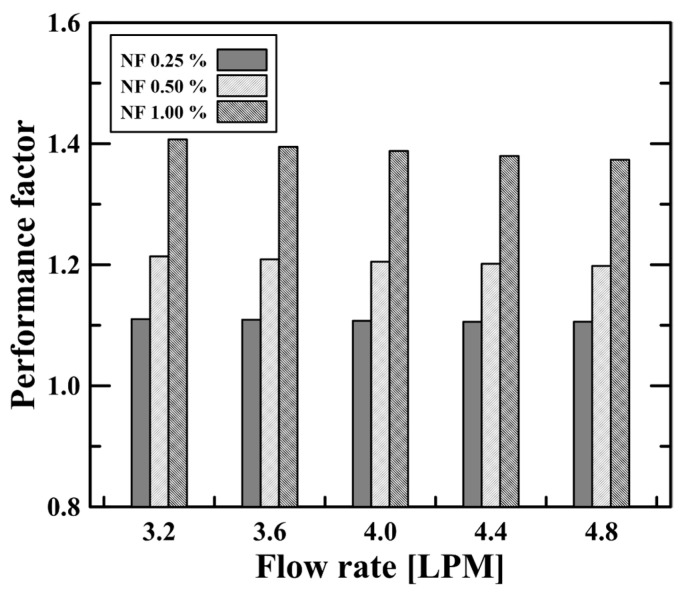
Performance factor of the nanofluids at different concentrations and under various flow rate conditions.

In this study, the nanofluid had a high Nusselt number (see Figure 7). Moreover, at a high concentration, there was an increased pressure drop (see Figure 8), i.e., the enhanced heat transfer of the nanofluids was accompanied by an increased pressure drop due to the large flow resistance from the high viscosity. The increase in the pressure drop may have an adverse effect from an economic perspective, as this will require greater pumping power. Therefore, it is necessary to consider both heat transfer characteristics (i.e., heat transfer coefficient and Nusselt number) and hydraulic characteristics (i.e., friction factor and pressure drop) if the nanofluids are to be employed as an enhanced working fluid. In this regard, we next evaluated the energy efficiency of the nanofluid by calculating the performance factor (PF) using the Nusselt number and pressure drop. The performance factor was calculated as follows [41,42,43,44]:

(22) $$\mathrm{PF}=\frac{Nu_{NF}/Nu_{w}}{{\left(f_{NF}/f_{w}\right)}^{1/3}}$$

(23) $$\frac{f_{NF}}{f_{w}}=\frac{\Delta P_{NF}/\rho_{NF}}{\Delta P_{w}/\rho_{w}}$$

where the NF and w subscripts represent the nanofluid and water, respectively. The performance factor describes how much the heat transfer performance improves relative to the increase in the pressure drop of the nanofluid. Figure 9 compares the performance factor at various flow rates and nanofluid concentrations. The performance factor increased with increasing concentration because the pressure drop and Nusselt number rose by a small amount (~3%) and a large amount (~143%) as the concentration increased to 1.0%. In other words, the increase in the heat transfer performance, which was much greater than the increase in the pressure drop, significantly improved the performance factor. The 1.0 vol.% nanofluid showed a performance factor of 1.4, while the 0.25 vol.% nanofluid had a performance factor of 1.1. Here, we emphasize that the performance factor evaluation presented in this study is limited to a specific heat transfer system, the double-pipe helical coil heat exchanger, and that other heat transfer systems will show different values depending on the kind of nanofluid, the size of the nanoparticle, and various concentration cases used. Nevertheless, the enhanced performance factors of the Al_2_O_3_ nanofluid are expected to provide the feasibility that the Al_2_O_3_ nanofluid has the potential to be used as an enhanced working fluid in many heat transfer applications. In conclusion, the heat transfer and hydraulic characteristics of the Al_2_O_3_/water nanofluid found using a two-phase numerical approach will be helpful as validation data in future studies.

## 4. Conclusions

This study numerically investigated the heat transfer and hydraulic characteristics of a water/Al_2_O_3_ nanofluid in a double-pipe helical coil heat exchanger. A two-phase Eulerian model was used to simulate the heat transfer coefficient, Nusselt number, and pressure drop for the various nanofluid concentrations (i.e., volume fraction, *φ*~1.0 vol.%) under different flow rate conditions. The main results can be summarized as follows:

(1) A comparison of the velocity profiles with different coil lengths (i.e., degrees) revealed that the flow in the double-pipe helical coil heat exchanger was fully developed after two rotations (i.e., 720 degrees). The flow and heat transfer characteristics (i.e., heat transfer coefficient, Nusselt number, and pressure drop) were accordingly evaluated after two rotations to reflect the fully developed flows.
(2) Convective heat transfer was enhanced as the flow rate of nanofluids increased, as indicated by the large heat transfer coefficient and Nusselt number. The heat transfer was also enhanced as the nanofluid concentration (i.e., volume fraction) rose. The 1.0 vol.% nanofluid (i.e., the highest concentration) showed a heat transfer coefficient 1.43 times and a Nusselt number 1.38 times greater than water due to the improved thermal properties (i.e., thermal conductivity).
(3) The pressure drop was significantly affected by the flow rate conditions. As the flow rate increased, the pressure drop increased due to the high dynamic pressure loss and enhanced turbulence in the curved pipe. Additionally, the pressure drop of the nanofluids that flowed through the heat exchanger was greater than that of water, with an increase rate of up to 3%. This was because of the enhanced flow resistance caused by the increased viscosity with an increased nanofluid concentration.
(4) The energy efficiency of the nanofluids was evaluated by calculating the performance factor based on the Nusselt number and pressure drop. The 1.0 vol.% nanofluid had a performance factor of 1.4 relative to water, which means that the Al_2_O_3_ nanofluids showed a much larger enhanced heat transfer performance compared to the increased pressure drop, so that the Al_2_O_3_ nanofluids can be used as an improved heat transfer fluid.

## Figures and Tables

**Table 1:** Detailed sizes of the double-pipe helical coil heat exchanger.

	Inner Tube (Hot Side)	Annulus Tube (Cold Side)
Inner diameter	0.00774 m	0.01340 m
Outer diameter	0.00952 m	0.01588 m
Coil diameter	0.1524 m	
Coil pitch	0.0159 m	
Turns	4.5	

**Table 2:** Properties of Al_2_O_3_ nanofluid components.

Component	Size [nm]	Density [kg/m^3^]	Specific Heat [J/kg·K]	Thermal Conductivity [W/m·K]
Al_2_O_3_	25	3970	750	46
Water	$f\left(T\right)=a+bT+cT^{2}+dT^{3}$ (see Table 3)			

**Table 3:** Coefficients of polynomial equation for the density, viscosity, and thermal conductivity of water. Here, the specific heat was assumed as constant because this value has a very small deviation (i.e., 0.04%) in the temperature range of 20~30 °C.

$f\left(T\right)\;=\;a\;+\;bT\;+\;cT^{2}\;+\;dT^{3}$				
Water	a	b	c	d
Density	−9.10863 × 10^1^	1.00043 × 10^1^	−2.95327 × 10^−2^	2.75649 × 10^−5^
Viscosity	2.07186 × 10^−1^	−1.91026 × 10^−3^	5.92043 × 10^−6^	−6.15166 × 10^−9^
Thermal conductivity	−1.85073 × 10^0^	1.86749 × 10^−2^	−4.73644 × 10^−5^	4.16113 × 10^−8^
Specific heat	Constant value of 4180 J/kg·K			

**Table 4:** Numerical method employed in the present study.

Solution Method	Model or Scheme
Turbulence model	Realizable k-epsilon
Near wall treatment	Enhanced wall treatment
Pressure-velocity coupling	PC-SIMPLE
Gradient	Least-Squares Cell-Based
Momentum	QUICK
Volume fraction	QUICK
Turbulent kinetic energy	Power law
Turbulent dissipation rate	Power law
Energy	Second order upwind

**Table 5:** Flow rate conditions employed in the present study.

Case No.	Flow Rate at Hot Side	Flow Rate on Cold Side
Case 1	4.8 LPM (Re 15,600~16,500)	7.2 LPM (Re 6600)
Case 2	4.4 LPM (Re 14,300~15,100)	
Case 3	4.0 LPM (Re 13,000~13,700)	
Case 4	3.6 LPM (Re 11,700~12,400)	
Case 5	3.2 LPM (Re 10,400~11,000)	

**Table 6:** Mesh conditions employed in the grid independence test.

Case No.	Y^+^	Cell/m^3^
Mesh case 1	6.50	4.8 × 10^10^
Mesh case 2	3.20	5.3× 10^10^
Mesh case 3	1.35	6.7× 10^10^
Mesh case 4	1.27	7.1× 10^10^

## Data Availability

The data presented in this study are available on request from the corresponding author.

## Nomenclature

*D*	Diameter (m)
*F*	Force (N)
*f*	Friction factor
*g*	Gravitational acceleration (m/s^2^)
*h*	Heat transfer coefficient (W/m^2^K)
*K*	Interface momentum transfer coefficient
*k*	Thermal conductivity (W/mK)
*Nu*	Nusselt number
*P*	Pressure (N/m^2^)
*PF*	Performance factor
*Pr*	Prandtl number
*Q*	Heat transfer rate (W)
*R*	Radius (m)
*r*	r-axis distance (m)
*Re*	Reynold number (-)
*T*	Temperature (°C)
*u*	Flow velocity (m/s)
*v*	Velocity (m/s)
*Greek letters*	
*μ*	Dynamic viscosity (kg/m∙s)
*ρ*	Density (kg/m^3^)
*φ*	Volume fraction
*Subscripts*	
*h*	Hydraulic diameter
*l*	Liquid phase
*lift*	Lift force
*NF*	Nanofluid
*s*	Solid phase
*vm*	Virtual mass force
*w*	Water
